# Fecal microbiota transplantation to maintain remission in Crohn’s disease: a pilot randomized controlled study

**DOI:** 10.1186/s40168-020-0792-5

**Published:** 2020-02-03

**Authors:** Harry Sokol, Cecilia Landman, Philippe Seksik, Laurence Berard, Mélissa Montil, Isabelle Nion-Larmurier, Anne Bourrier, Guillaume Le Gall, Valérie Lalande, Alexis De Rougemont, Julien Kirchgesner, Anne Daguenel, Marine Cachanado, Alexandra Rousseau, Élodie Drouet, Michelle Rosenzwajg, Hervé Hagege, Xavier Dray, David Klatzman, Philippe Marteau, Saint-Antoine IBD Network, Lionel Arrivé, Laurent Beaugerie, Anne Bourrier, Marine Camus, Najim Chafai, Édouard Chambenois, Ulriikka Chaput, Chloé Martineau, Laurence Monnier-Cholley, Clotilde Debove, Xavier Dray, Jean-François Fléjou, Nadia Hoyeau, Julien Kirchgesner, Cecilia Landman, Jérémie H. Lefèvre, Philippe Marteau, Isabelle Nion-Larmurier, Violaine Ozenne, Yann Parc, Philippe Seksik, Harry Sokol, Magali Svrcek, Laurent Beaugerie, Tabassome Simon

**Affiliations:** 1 Centre de Recherche Saint-Antoine, CRSA, AP-HP, Hôpital Saint Antoine, Service de Gastroenterologie, Sorbonne Université, Inserm, 75012 Paris, France; 2 Department of Gastroenterology, Saint Antoine Hospital, Assitance Publique-Hopitaux de Paris (APHP), 184 rue du Faubourg Saint-Antoine, 75571 Paris, CEDEX 12 France; 3 INRA, UMR1319 Micalis & AgroParisTech, Jouy en Josas, France; 4 French Group of Fecal Transplantation (GFTF), Paris, France; 5 Inflammation-Immunopathology-Biotherapy Department (DHU i2B), Paris, France; 6 Clinical Research Platform (URC-CRC-CRB), AP-HP Saint-Antoine Hospital, Paris, France; 7 Department of Microbiology, Saint Antoine Hospital, Assitance Publique-Hopitaux de Paris (APHP), Paris, France; 8 National reference center for enteric virus, Virology laboratory, CHU de Dijon, France; UFR des Sciences de Santé, Université de Bourgogne, Dijon, France; 9 Department of Pharmacy, Saint Antoine Hospital, Assitance Publique-Hopitaux de Paris (APHP), Paris, France; 10 Immunology-Immunopathology-Immunotherapy (I3), Sorbonne University-UPMC Univ Paris 06, INSERM UMR S959, 75005 Paris, France; 11 Biotherapy (CIC-BTi), Pitié- Salpêtrière Hospital, AP-HP, 75013 Paris, France; 12 Department of Gastroenterology, CHI Créteil, Créteil, France; 13 Department of Hepato-Gastroenterology, APHP, Saint Antoine Hospital, Sorbonne University, Paris, France; 14 Department of Clinical Pharmacology, APHP, Saint Antoine Hospital, Paris, France

**Keywords:** Fecal microbiota transplantation, Crohn’s disease, Randomized controlled trial

## Abstract

**Background:**

The role of the gut microbiota in Crohn’s disease (CD) is established and fecal microbiota transplantation (FMT) is an attractive therapeutic strategy. No randomized controlled clinical trial results are available. We performed a randomized, single-blind, sham-controlled pilot trial of FMT in adults with colonic or ileo-colonic CD.

**Method:**

Patients enrolled while in flare received oral corticosteroid. Once in clinical remission, patients were randomized to receive either FMT or sham transplantation during a colonoscopy. Corticosteroids were tapered and a second colonoscopy was performed at week 6. The primary endpoint was the implantation of the donor microbiota at week 6 (Sorensen index > 0.6).

**Results:**

Eight patients received FMT and nine sham transplantation. None of the patients reached the primary endpoint. The steroid-free clinical remission rate at 10 and 24 weeks was 44.4% (4/9) and 33.3% (3/9) in the sham transplantation group and 87.5% (7/8) and 50.0% (4/8; one patient loss of follow-up while in remission at week 12 and considered in flare at week 24) in the FMT group. Crohn’s Disease Endoscopic Index of Severity decreased 6 weeks after FMT (*p* = 0.03) but not after sham transplantation (*p* = 0.8). Conversely, the CRP level increased 6 weeks after sham transplantation (*p* = 0.008) but not after FMT (*p* = 0.5). Absence of donor microbiota engraftment was associated with flare. No safety signal was identified.

**Conclusion:**

The primary endpoint was not reached for any patient. In this pilot study, higher colonization by donor microbiota was associated with maintenance of remission. These results must be confirmed in larger studies **(**NCT02097797).

Video abstract.

## Background

Crohn’s disease (CD) is a chronic relapsing inflammatory bowel disease (IBD). Its pathogenesis is not fully understood, but it is now acknowledged that it is related to an abnormal activation of the gastro-intestinal immune system towards the gut microbiota in genetically susceptible hosts and under the influence of environmental factors [1]. Notably, many studies have shown that the intestinal microbiota in Crohn’s disease patients is abnormal and unbalanced when compared to non-IBD controls, with an increased proportion of potentially pro-inflammatory bacteria such as *Escherichia coli* and a decrease in anti-inflammatory bacteria such as *Faecalibacterium prausnitzii* [2, 3]. Moreover, several studies in mice suggest that this dysbiotic microbiota might play an active role in triggering or worsening the inflammatory process [4, 5].

Current therapeutic strategies aim at inhibiting the over-activated immune system and largely ignore the microbial component of disease pathogenesis. Conventional immunosuppressive treatments and biologics used in CD are expensive and associated with potentially severe complications such as infections [6, 7] and cancers, [8] justifying the need for other innovative approaches.

Fecal microbiota transplantation (FMT) is now recommended in guidelines for treating recurrent *Clostridium difficile* infection [9–11]. Although the pathogenesis involved in CD is different, FMT is a potential therapeutic strategy since transferring a healthy microbiota to a CD patient could restore the appropriate host-microbiota crosstalk.

No randomized controlled study evaluating FMT in CD patients has been published to date, but heterogeneous cases or small series, as well as small open-label uncontrolled studies, have been reported and suggest a beneficial effect for the induction of clinical remission [12–15]. Most of the available data regarding FMT in IBD is in ulcerative colitis (UC). The efficacy of FMT in patients with UC has been evaluated in four published randomized controlled studies, all of which aimed at inducing remission. Three studies demonstrated the superiority of FMT compared to placebo [16–18] and one did not show significant differences between the two groups studied [19]. However, even in the studies that demonstrated superiority of FMT, the difference compared with placebo was moderate, with 24, 27, and 32% remission rates versus 5, 8 and 9% at 7 and 8 weeks, respectively, despite multiple FMT administrations. Patients received FMT enemas once a week for 6 weeks in the first study [16], 1 FMT via colonoscopy, followed by 5 FMT enemas per week for 8 weeks in the second study [17] and 1 FMT via colonoscopy, followed by 2 FMT enemas over 7 days in the third study [17]. Moreover, each FMT was derived from 3 to 7 donors in the second study and from 3 to 4 donors in the latter study. These results suggest that targeting only the gut microbiota might not be sufficient in IBD. Moreover, the ideal time to perform an FMT in IBD is not known, although there are some arguments to suggest that performing an FMT in patients who have already achieved remission might be more appropriate than during an active flare, particularly in Crohn’s disease [20]. As the gut microbiota is dramatically altered by intestinal inflammation, a healthy microbiota transferred to an inflamed gut might be rapidly altered, thus limiting its potential therapeutic effect. Furthermore, transferring a massive quantity of microbes into an inflamed gut with epithelial barrier disruption could potentially have detrimental effects by stimulating inflammation and allowing bacterial translocation. This is of particular relevance in CD, a condition that is characterized by transmural inflammation.

Here, we performed the first randomized controlled study evaluating FMT in CD. We chose an original strategy, targeting both immune and microbiota components of the disease pathogenesis, in which a single FMT or sham transplantation was performed in patients in whom clinical remission had been achieved using corticosteroids.

## Materials and methods

### Study design

We conducted a multicenter (*n* = 6), randomized, single-blind placebo-controlled trial of fecal microbiota transplantation (FMT) in patients with colonic or ileo-colonic Crohn’s disease who achieved remission with corticosteroids. In the FMT group, patients received a single fecal transplant (fecal microbiota from one healthy donor) after clinical remission was obtained using oral corticosteroids. The fecal microbiota consisted of 50–100 g of stool from a donor resuspended in 250–350 ml of sterile sodium chloride and filtered. After colon cleansing (4 l of polyethylene glycol), this fecal preparation, or the vehicle (physiological serum) in the sham group, was infused into the cecum during a colonoscopy.

The sponsor of the study was Assistance Publique–Hôpitaux de Paris (Department for Clinical Research and Innovation).

### Participants

Adult patients (aged 18–70 years) with health insurance who had Crohn’s disease with colonic or ileo-colonic involvement were recruited between August 26, 2014, and February 14^,^ 2017, from the Departments of Gastroenterology of Saint Antoine Hospital (Paris, France), Lariboisiere Hospital (Paris, France), Henri Mondor Hospital (Creteil, France), Saint Louis Hospital (Paris, France), Centre Hospitalier Intercommunal (Creteil, France), and Montfermeil Hospital (Montfermeil, France).

Patients were not eligible if there was active fistulizing disease; perianal or abdominal abscesses; complications requiring surgical treatment; treatment with anti-tumor necrosis factor (TNF) agents (ongoing or stopped in the month preceding randomization); immunosuppressant treatment started or stopped in the 3 months preceding randomization; intake of non-steroidal anti-inflammatory drugs (NSAIDs) in the 4 weeks preceding randomization; antibiotic or antifungal treatment in the 4 weeks preceding colonoscopy; probiotics intake in the 4 weeks preceding colonoscopy; *Clostridium difficile* infection in the 10 days preceding randomization; and any contraindication to colonoscopy or anesthesia and pregnancy.

All the patients except one were recruited in St Antoine Hospital (the last one was recruited in Lariboisiere Hospital). The FMT or sham, as well as the follow-up, was performed for all patients in Saint Antoine Hospital (Paris, France).

Patients with active disease at screening (defined as a Harvey Bradshaw Index > 4) were selected and treated orally with prednisolone (minimum dose 40 mg/day, maximum dose 1 mg/kg/day). Patients who achieved clinical remission within the 3 weeks following the commencement of corticosteroids (defined as a Harvey Bradshaw Index < 5) were randomized to receive either FMT or sham FMT by colonoscopy. The maximum time between starting corticosteroids and FMT or sham FMT was 5 weeks. Corticosteroids were then tapered according to a pre-defined schedule: decrease by 10 mg every week until 50% of the initial dose and then decrease by 5 mg per week until complete cessation. All patients had a clinical assessment and biochemical evaluation at weeks (W) 2, W6, W10, W14, W18, and W24 after the intervention and a second colonoscopy at W6 (Additional file 1). Clinical activity was evaluated with the Crohn’s disease activity index (CDAI) and Harvey Bradshaw Index (HBI). Biological analysis included blood C reactive protein (CRP) level and fecal calprotectin level. Endoscopic lesions were scored using the Crohn’s Disease Endoscopic Index of Severity (CDEIS).

### Donors

We recruited healthy donors by advertisement in an authorized biomedical research location, targeting healthy volunteers, outlining donor visits, and based upon the following criteria:

- Age > 20 years and < 50 years
- 27 kg/m^2^ > body mass index > 17 kg/m^2^ (body mass index is defined as the body mass divided by the square of the body height)
- Regular bowel movements with usually one bowel movement in the morning
- Health insurance

Donor screening was performed according to the French National Security Drugs Agency (ANSM) recommendations (see Additional file 2 for details). According to these recommendations, the screening test results were valid for 2 weeks, corresponding to the authorized donation period.

Of the 213 donors who were initially prescreened by telephone, 64 underwent the clinical and biological screening and five ultimately served as study donors. As FMT was performed with fresh stool, three donors prescreened for CMV, EBV and toxoplasmosis status were selected and screened according to ANSM recommendations for each patient in the FMT arm. On the day of the planned FMT, one to three donors with valid screening tests were convened at the hospital for stool donation. In total, five different donors were used. Each FMT was prepared freshly from one single donor. One donor was used for three recipients, one donor was used for two recipients, and the three last donors were used for a single recipient each.

### Sample preparation

On the day of the planned FMT, fresh stool from donors, kept at 4 °C, was used within 6 h of emission. Fifty to 100 g of stool was resuspended in 250–350 ml of sterile sodiume chloride (0.9% NaCl), homogenized in a single usage blender and filtered using sterile, non-woven gauze. The solution was adjusted to a final volume of 300 ml with sterile saline and transferred to five 60-ml syringes for administration through the operating channel of the colonoscope during colonoscopy. All the preparation procedures were performed in a biological safety cabinet.

### Outcomes

The primary outcome was the successful colonization of the donor microbiota at 6 weeks. Colonization was defined as being successful if the fecal microbiota of the recipient 6 weeks after FMT was more similar to the fecal microbiota of the donor than to the recipient before FMT (Sorensen’s index [recipient 6 weeks after FMT vs donor] > Sorensen’s index [recipient 6 weeks after FMT vs recipient before FMT]). Moreover, it was required that the similarity (assessed by Sorensen’s index) between donor and recipient at 6 weeks was ≥ 0.6.

Secondary endpoints were the feasibility of the FMT procedure (frequency of evaluable patients in each group) and the clinical flare rate in the 24 weeks following FMT. A clinical flare was defined as a CDAI > 220 points, by a CDAI between 150 and 220 with an increase > 70 compared with baseline, or by the need for surgery or to start a new medical treatment for CD. Flare outcome was analyzed in the intention to treat population throughout the paper. CRP, leukocyte level, fecal calprotectin, CDEIS, fecal microbiota composition, and diversity were also compared among groups.

### Fecal sample collection

Fecal samples were longitudinally collected from patients, 2 weeks before FMT (W2), just before FMT (D0) and then at W2, W6, W10, W14, W18, and W24. The fecal samples were freshly collected by the patient in a sterile container and kept at 4 °C in an anaerobic atmosphere using an Anaerocult® system until storage at − 80 °C, 6 h maximum after a bowel movement.

### Sample size

We hypothesized that at least 80% of the recipients in the FMT group would achieve fecal transplantation success compared to 0.5% in the control group (sham transplantation), using a bilateral test, with alpha = 5% and beta = 10%. Considering 50% of non-evaluable patients, 24 patients (12 in each group) were estimated to be required for inclusion.

### Randomization

Patients were randomized in a 1:1 ratio. Centralized block randomization was performed by an independent statistician from the clinical research platform (URC-Est) and the size of the blocks was not communicated to the investigator.

### Ethics

The study was performed in accordance with the requirements of Good Clinical Practice and the Revised Declaration of Helsinki. The study was registered on www.clinicaltrials.gov (NCT02097797). All participants provided written informed consent to participate after receiving verbal and written information about the study. The protocol was approved by the Ethics committee (Comite de Protection des Personnes, CPP), on July 17, 2013. The final authorization was obtained a year later from the French National Security Drug Agency (Agence National pour la Sécurité du Médicament).

### Gut microbiota analysis

Fecal genomic DNA was extracted from 200 mg of feces as previously described [4]. Following microbial lysis with both mechanical and chemical steps, nucleic acids were precipitated in isopropanol for 10 min at room temperature, incubated for 15 min on ice, and centrifuged for 30 min at 15,000*g* and 4 °C. Pellets were suspended in 112 μL of phosphate buffer and 12 μL of potassium acetate. After RNase treatment and DNA precipitation, nucleic acids were recovered via centrifugation at 15,000*g* and 4 °C for 30 min. The DNA pellet was suspended in 100 μL of TE buffer.

Microbial diversity and composition were determined for each sample by targeting a portion of the ribosomal genes. A 16S rRNA gene fragment comprising V3 and V4 hypervariable regions (16S; 5′-TACGGRAGGCAGCAG-3′ and 5′-CTACCNGGGTATCTAAT-3′) was amplified using an optimized and standardized 16S-amplicon-library preparation protocol (Metabiote, GenoScreen). Briefly, 16S rRNA gene PCR was performed using 5 ng genomic DNA according to the manufacturer’s protocol (Metabiote) using 192 bar-coded primers (Metabiote MiSeq Primers, GenoScreen) at final concentrations of 0.2 μM and an annealing temperature of 50 °C for 30 cycles. The PCR products were purified using an Agencourt AMPure XP-PCR Purification system (Beckman Coulter), quantified according to the manufacturer’s protocol, and multiplexed at equal concentrations. Sequencing was performed using a 250-bp paired-end sequencing protocol on an Illumina MiSeq platform (Illumina) at GenoScreen. Raw paired-end reads were subjected to the following process: (1) quality filtering using the PRINSEQ-lite PERL script [21] by truncating the bases from the 3′ end that did not exhibit a quality < 30 based on the Phred algorithm; (2) paired-end read assembly using FLASH (fast length adjustment of short reads to improve genome assemblies) with a minimum overlap of 30 bases and a 97% overlap identity; and (3) searching and removing both forward and reverse primer sequences using CutAdapt, with no mismatches allowed in the primers sequences. Assembled sequences for which perfect forward and reverse primers were not found were eliminated.

The sequences were demultiplexed and quality filtered using the Quantitative Insights Into Microbial Ecology (QIIME, version 1.9.1) software package [22], and the forward and reverse Illumina reads were joined using the fastq-join method (http://code.google.com/p/ea-utils). Following an open reference picking strategy, the sequences were assigned to OTUs using the UCLUST algorithm [23] with a 97% threshold of pairwise identity and classified taxonomically using the Silva reference database (version 132) [24]. Principal coordinate analyses of the Bray Curtis distance were performed and used to assess the variation between groups (beta diversity). Significance was assessed using ANOSIM (9999 permutations). The Shannon and Chao1 diversity indices were calculated using rarefied data (depth = 29,000 sequences/sample) and used to characterize species diversity in a community. Raw sequence data (joined sequences) are accessible in the European Nucleotide Archive (accession number PRJEB33031) and the corresponding metadata file is available in Additional file 3. Sourcetracker, a Bayesian approach, was used to identify sources and proportions of OTUs in the different samples [25]. Differential analysis was performed using the linear discriminant analysis effect size (LEfSe) pipeline [26].

### Statistical analysis

The statistical analysis followed the intention-to-treat approach and results were reported according to the CONSORT statement recommendations.

Patients’ characteristics were described globally and per group of randomization. Qualitative data were expressed as number (%) and quantitative data were expressed as medians and interquartile range (IQR). The primary outcome was compared between groups with a Fisher’s exact test. One primary outcome was missing at 6 weeks (one patient was lost to follow-up) and was replaced by failure. Quantitative variables were compared using a non-parametric Wilcoxon rank-sum tests.

The similarity of the fecal microbiota samples was assessed using the Sorensen similarity index (Sorensen similarity index = [1 – Bray Curtis dissimilarity index]). The non-parametric Wilcoxon rank-sum test was used to compare the similarity indices between groups. Exploratory analysis compared survival without clinical flare until W24 within groups.

A graphical representation of the probability of remaining in remission was drawn using the Kaplan-Meier method. The log-rank test was used to compare survival curves. All tests were two-sided. *p* values < 0.05 were considered statistically significant. SAS® 9.3 software (SAS Institute, Cary, NC, USA) was used for statistical analyses. Prism 7.04 (GraphPad, La Jolla, CA, 92037, USA) was used to make graphics.

## Results

### Patient characteristics and primary outcome

**Fig. 1:**
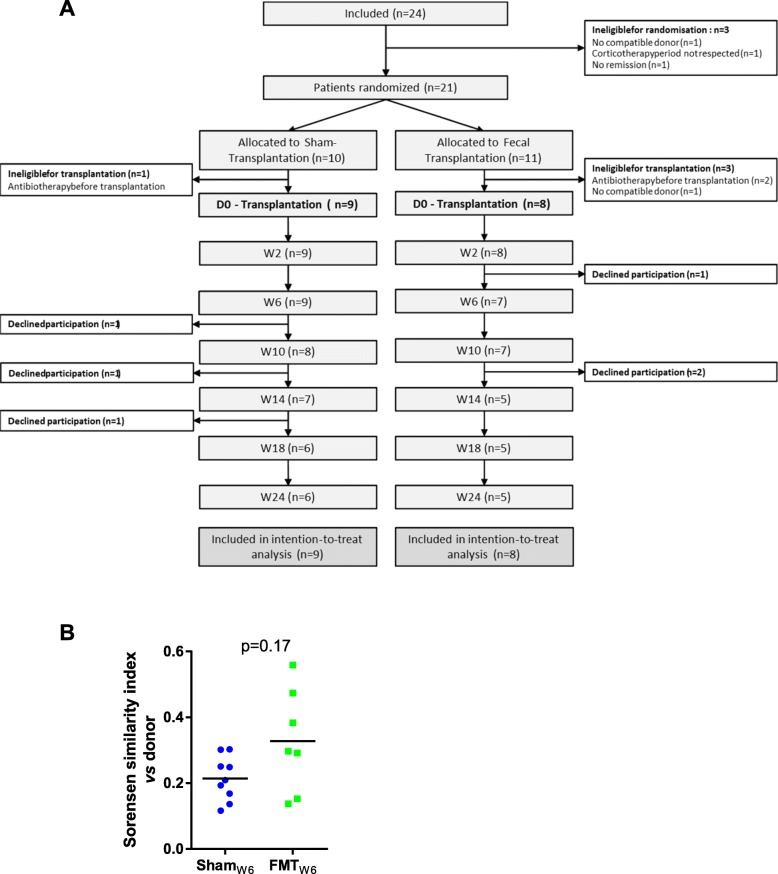
Flow chart of patients and primary endpoint. **a** Flow chart of patients included and excluded from analysis, according to CONSORT, Consolidated Standards of Reporting Trials. **b** Sorensen similarity index between donor and recipient fecal microbiota 6 weeks after FMT or sham. For **b**, the Wilcoxon rank-sum test was used

Overall, 24 patients were included in the study and 21 patients were randomized. Among the 10 and 11 patients allocated respectively to the sham and to the fecal transplantation groups, 9 and 8 went on to receive the sham and fecal transplantation (included in the intention to treat analysis); 3 patients were ineligible because of antibiotic intake (these patients were found positive for *C. difficile* or *Dientamoeba fragilis* following their initial screen) and one because no compatible donor was identified (Fig. 1a). The characteristics of the two groups analyzed at the time of intervention are shown in Table 1 (characteristics of patients at selection before starting corticosteroids are presented in Additional file 4). Six patients (3 in each group) dropped out while in the study, with no reason given. Four of them experienced a flare prior to dropping out. For one patient, clinical follow-up was obtained by contacting the patient and his physician. The last patient dropped out while in remission at week 12 and was considered in flare at week 24.

**Table 1:** Characteristics of patients at baseline (time of intervention)

	Whole population (*n* = 17)	Sham transplantation (*n* = 9)	Fecal transplantation (*n* = 8)	*p* value (sham vs FMT)
Age (years)	34.0 [29.0; 37.0]	34.0 [33.0; 52.0]	31.5 [27.5; 36.5]	0.10
Male	9 (52.9)	4 (44.4)	5 (62.5)	0.64
Female	8 (47.1)	5 (55.6)	3 (37.5)	
BMI (kg/m^2^)	22.3 [19.8; 23.3]	22.7 [22.3; 23.3]	21.0 [18.6; 22.9]	0.15
Smoking, never/past/active	6 (35.3)/6 (35.3)/5 (29.4)	3 (33.3)/4 (44.4)/2 (22.2)	3 (37.5)/2 (25.0)/3 (37.5)	0.7
Disease duration (years)	9 [5; 15]	11 [8; 15]	8.5 [4; 12.8]	0.19
Montreal, L2/L3	7 (41.2)/10 (58.8)	5 (55.6)/4 (44.4)	2 (25.0)/6 (75.0)	0.33
Hemoglobin (g/L)	138 [121; 142]	138 [121; 142]	134 [116; 147]	0.46
White cell count (10^9^/L)	10.9 [9.3; 12.8]	12.4 [10.0; 13.3]	10.1 [8.6; 11.3]	0.06
Platelet (10^9^/L)	338.0 [278.0; 405.0]	346.0 [280.0; 385.0]	292.5 [275.5; 493.5]	0.42
CRP (mg/L)	3.0 [3.0; 3.1]	3.0 [3.0; 4.2]	3.0 [3.0; 3.0]	0.31
Albumin (g/L)	40.7 [39.4; 42.7]	40.5 [39.4; 42.5]	41.4 [39.6; 44.0]	0.27
Fecal calprotectin (ug/g)	102.0 [40.0; 157.0]	117.0 [45.0; 177.3]	53.5 [38.0; 112.0]	0.22
Previous azathioprine treatment	10 (58.8)	6 (66.6)	4 (50.0)	0.49
Previous anti-TNF therapy	5 (29.4)	3 (33.3)	2 (25.0)	0.71
Previous other biological therapy	3 (17.7)	2 (22.2)	1 (12.5)	0.60
History of intestinal resection	2 (11.8)	1 (11.1)	1 (12.5)	0.92
HBI score	2.0 [0.0; 3.0]	2.0 [0.0; 3.0]	1.5 [0.5; 2.5]	0.35
CDAI score	62.0 [41.0; 109.0]	54.0 [35.0 ; 103.0]	65.5 [51.5; 173.5]	0.16
CDEIS score	4.6 [0.2; 10.5]	1.6 [0.0; 6.0]	7.1 [3.5; 12.2]	0.062

Categorical parameters indicated as *n* (%) and continuous values indicated as P50 [P25; P75]. Quantitative variables were compared using a non-parametric Wilcoxon rank-sum tests. Qualitative variables were compared using Fisher’s exact test

*BMI* body mass index, *CRP* C reactive protein, *HBI* Harvey Bradshaw Index, *CDAI* Crohn’s disease activity index, *CDEIS* Crohn’s Disease Endoscopic Index of Severity

Thirteen serious adverse events were reported, including 9 CD flares (6 in the sham group and 3 in the FMT group). Among the 6 others, there included one episode of gastroenteritis, one episode of food poisoning, one case of transient asthenia, and one cutaneous abscess reported in the FMT group, while one case of shoulder fracture and one case of decreased visual acuity were reported in the sham group. None of the adverse events were considered to be related to the FMT.

None of the subjects included in the study achieved the primary endpoint regarding the colonization of the donor microbiota at 6 weeks as defined in the protocol, as the donor-recipient Sorensen’s index at W6 was below 0.6 in all patients (Fig. 1b). However, the judgment criterion was defined in 2012, in a period where no reference was available on the topic and was thus arbitrary. All the analyses thus focused on secondary endpoints.

### Efficacy in maintaining remission

**Fig. 2:**
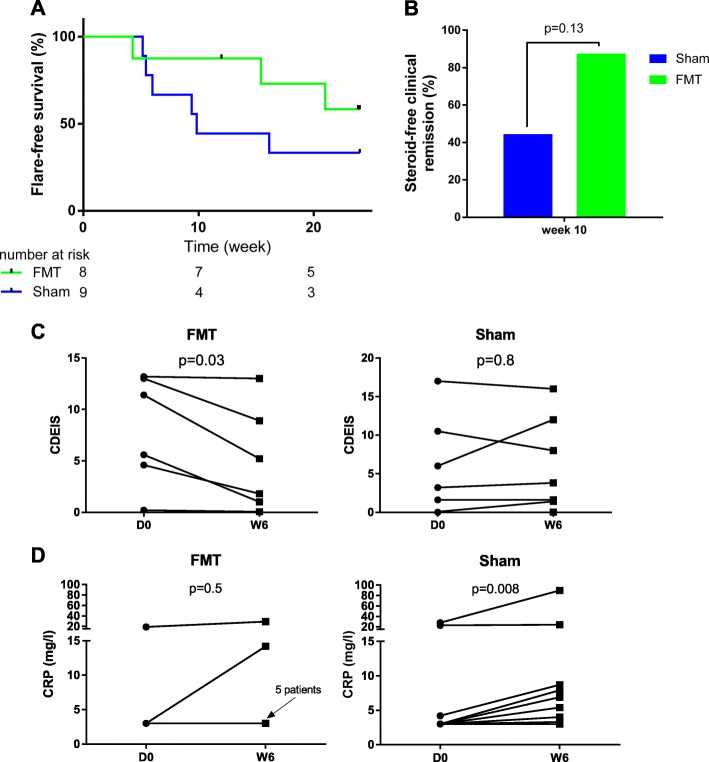
Clinical efficacy of FMT in CD patients who achieved clinical remission with steroids. **a** Flare-free survival of patients in the FMT and the sham groups compared using log-rank test. **b** Steroid-free remission at week 10 after FMT or sham transplantation compared with Fisher’s exact test. Change in CDEIS (**c**) and CRP (**d**) between day 0 and week 6 for FMT and sham treatment groups, evaluated with the paired Wilcoxon test. One patient in each group was not evaluable for CDEIS because of a bowel-cleansing problem at week 6. One sample was not available for CRP in the FMT group at week 6

**Fig. 3:**
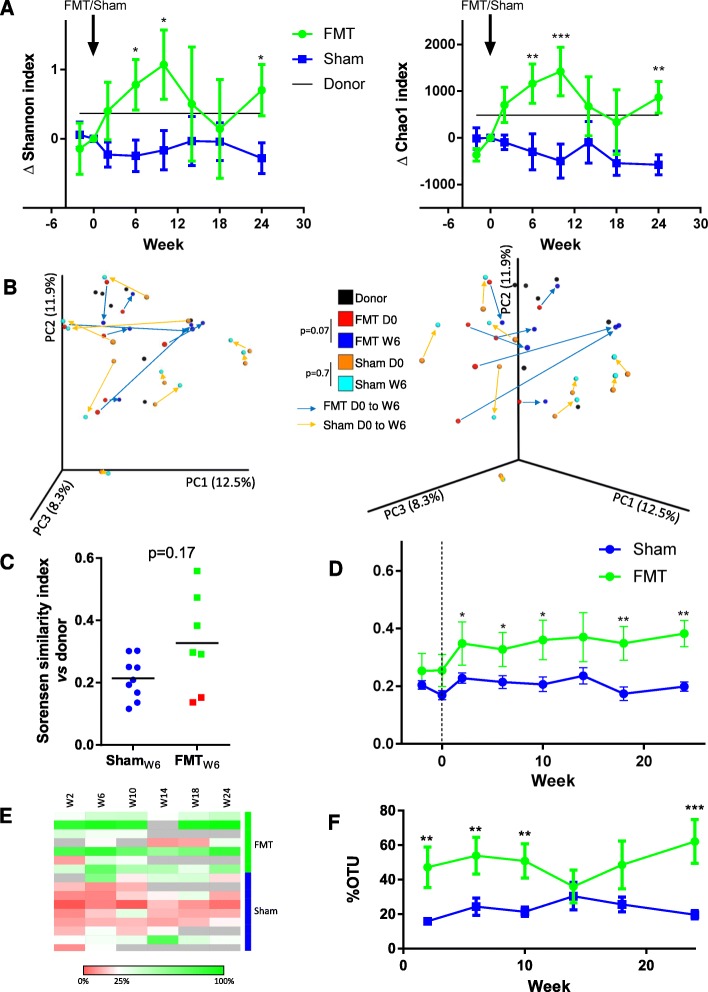
Effect of FMT on fecal microbiota composition in recipients. **a** Change in Shannon and Chao1 alpha diversity indices compared to day 0. For donors, the change was calculated using the mean of the FMT group. **b** Principal coordinate analysis of Bray-Curtis distance. PC1, PC2, and PC3 represent the top three principal coordinates that captured most of the variance. Arrows connect samples from the same patient before (day 0) and 6 weeks after FMT or sham. Two different view angles of the same PCoA plot are shown for clarity. Groups were compared using the ANOSIM method (9999 permutations). **c** Sorensen similarity index between donor and recipient fecal microbiota 6 weeks after FMT or sham. Red dots are considered as “FMT failure.” For the sham group, the mean of the Sorensen with each donor was indicated. **d** Evolution of Sorensen similarity index between donor and recipient fecal microbiota. **e**, **f** Proportion of different OTUs absent in samples before FMT or sham (W2 and D0) and present after FMT or sham. For **a**, **d**, and **f**, two-way Anova with *q* < 0.1, according to a Benjamini-Hochberg FDR, was used. For **c**, the Wilcoxon rank-sum test was used. *p < 0.05; **p < 0.01; ***p < 0.001

The incidence of flare was lower in the FMT than in the sham group but the difference did not reach statistical significance (log-rank test, *p* = 0.23, steroid-free clinical remission at week 10: 87.5% (7/8) in the FMT group vs 44.4% (4/9) in the sham group, Fig. 2a, b). Among the three patients who experienced a flare in the FMT arm, two were treated with adalimumab and no data was available for the third patient. Among the six patients who experienced flare in the sham arm, one received ustekinumab, one received corticosteroids and azathioprine, three received corticosteroids, and no data was available for the last patient. The CDEIS decreased significantly 6 weeks after FMT (8.5 [4.6; 13.0] vs. 3.5 [1.0; 8.9]; *p* = 0.03) but not after sham (2.4 [0.0;8.3] vs. 2.7 [0.7;10.0]; *p* = 0.8; Fig. 2c). Of note, one patient in each group was not evaluable for CDEIS at week 6 because of a bowel-cleansing problem and was thus not taken into account for this specific analysis. Moreover, the lower CDEIS score at baseline in some patients of the sham group might limit the possibility to observe a further decrease. Similarly, 6 weeks after FMT, CRP levels remained stable (3.0 [3.0; 3.0] vs. 3.0 [3.0; 14.2] mg/l; *p* = 0.5) while they had already started to increase in the sham group (3.0 [3.0; 4.2] vs. 6.9 [4.0; 8.7] mg/l; *p* = 0.008; Fig. 3d). Although some trends were observed, no statistically significant differences were observed between D0 and W6 in the FMT and sham groups regarding HBI, CDAI, and fecal calprotectin (Additional file 5 A–C). Neutrophil counts decreased in both groups, but this was significant only in the FMT group (Additional file 5 D). The number of subjects included was too low to reach statistical significance in several secondary endpoints. Based on the 25% difference observed between the intervention and control groups regarding the relapse rate at W24, 96 or 122 patients would be required to achieve sufficient statistical power for unilateral and bilateral tests, respectively (with alpha = 5% and beta = 20%).

### FMT does not systematically induce shifts in microbiota composition

The gut microbiota composition and diversity was assessed in donors and patients at each visit. Alpha diversity was evaluated using the Shannon and Chao1 indices. We observed a significant increase in alpha diversity following FMT but not sham (Fig. 3a). However, this change was transient and alpha diversity returned to its initial level 14 weeks after FMT. When analyzed globally, beta diversity showed a trend towards a change in the microbiota profile between D0 and W6 in the FMT (*p* = 0.07) but not the sham (*p* = 0.7) group (Fig. 3b).

We then looked at the similarity between donor and recipient microbiota using the Sorensen index. No statistically significant difference was observed between the FMT and sham group at week 6 (Fig. 3c). However, for two patients in the FMT group, the Sorensen similarity index was particularly low at week 6 (0.12 and 0.15, respectively) compared to the other patients (0.29 to 0.56). This means that the donor microbiota poorly colonized these two patients, who can thus be considered as FMT failures. When excluding these two patients, a difference between the FMT and sham groups was clearly apparent (Additional file 6 A). In the patients with FMT failure, the increase in alpha diversity was shorter than that observed in the patients with FMT success (Additional file 6 B). Interestingly, colonization by the donor microbiota seemed to persist over time, as indicated by the relative stability of donor-patient similarity until the end of the follow-up period (Fig. 3d). A similar signal was observed when looking at OTUs that appeared after FMT or sham transplantation, as the abundance of these OTUs persisted in recipients throughout the follow-up period (Fig. 3e, f).

**Fig. 4:**
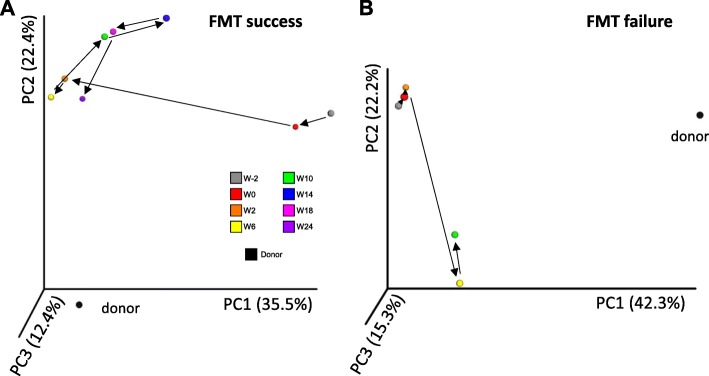
Evolution of the fecal microbiota beta-diversity in patients with successful or unsuccessful colonization by the donor microbiota. Principal coordinate analysis of Bray-Curtis distance in one patient with FMT success (**a**) and one patient with FMT failure (**b**). PC1, PC2, and PC3 represent the top three principal coordinates that captured most of the diversity. The fraction of diversity captured by the coordinate is given as a percentage

**Fig. 5:**
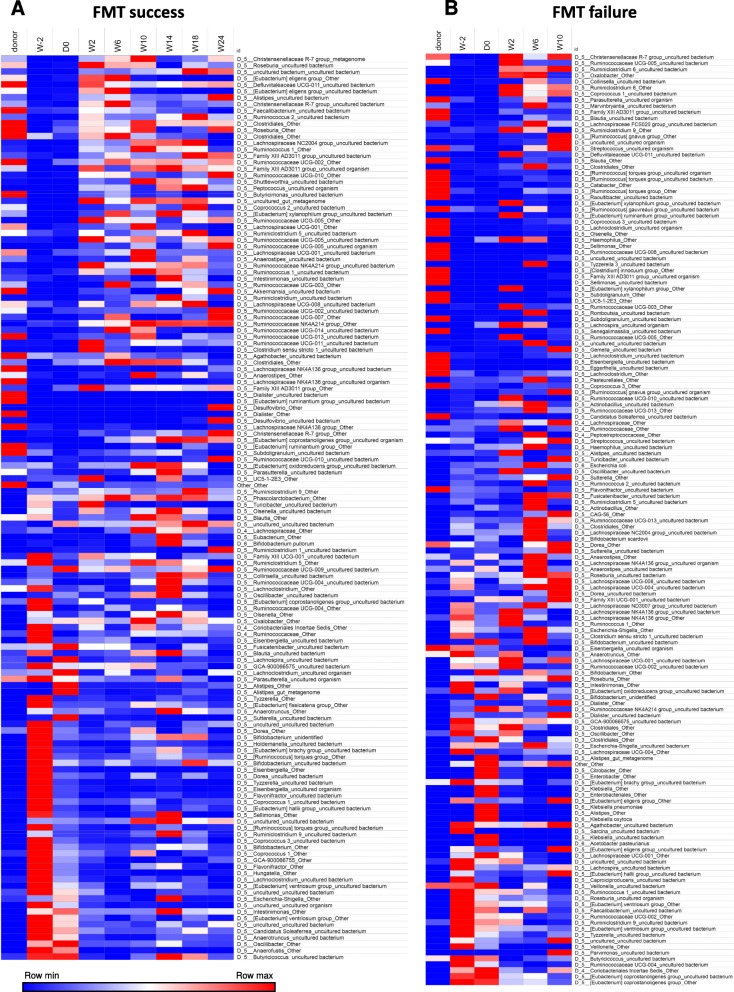
Evolution of the fecal microbiota bacterial taxa with successful or unsuccessful colonization by the donor microbiota. Abundance of bacterial taxa (at the genus/species level) during the follow-up period in one patient with FMT success (**a**) and one patient with FMT failure (**b**). Only taxa representing > 0.1% of the microbiota were taken into account in the analysis

We then studied the patients individually to better explore differing behaviors of the microbiota following transplantation into different recipients. The evolution of the fecal microbiota of one patient with FMT success and one patient with FMT failure are shown in Figs. 4 and 5. The same data for the other patients are shown in Additional files 7 and 8. In a patient with successful engraftment of the donor microbiota, there is a dramatic shift of the patient’s microbiota towards the donor’s microbiota after FMT. This change remains relatively stable during the follow-up period (Fig. 4a). Conversely, in a patient with FMT failure, the microbiota does not change between W0 and W2 and continues to follow an erratic path without getting closer to the donor’s microbiota at any subsequent time point (Fig. 4b). The abundance of bacterial taxa after FMT is shown in Fig. 5 and in Additional file 8. We used SourceTracker to identify the OTUs from donors that were found in recipients after FMT. Although the low number of patients did not allow us to demonstrate statistically significant differences, the proportion of donors’ OTUs at W2 and W6 was higher in patients with FMT success than in those with FMT failure (Additional file 8 C). Similar results were observed when using unweighted Unifrac similarity index (Additional file 9).

Finally, we looked at the clinical outcome according to FMT success or failure. The two patients who failed to be colonized by the donor microbiota exhibited an early flare, similar to the sham transplantation group. Conversely, patients with successful donor microbiota engraftment experienced a very low flare rate (log-rank test FMT vs sham group, *p* = 0.054, Additional file 10 A). Their steroid-free clinical remission rate at week 10 was higher than the sham group (100% vs 44.4%, *p* = 0.04, Additional file 10 B).

**Fig. 6:**
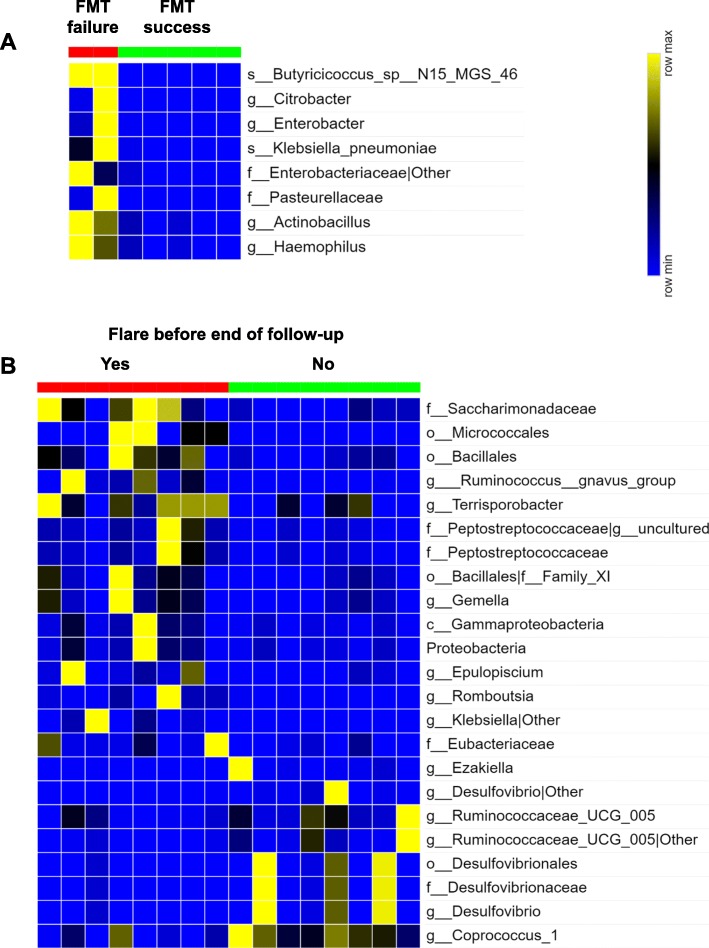
Microbial predictors of clinical outcome. **a** Bacterial taxa differentially enriched at baseline (day 0) in patients with FMT success and FMT failure (generated using LeFSE analysis). **b** Bacterial taxa at week 6 in the whole study population associated with flare vs no flare before the end of follow-up. Results were generated using LeFSE analysis and only statistically significant (linear differential analysis scores > 2, *p* < 0.05) taxa are shown

We then looked for microbial factors that could explain differences between FMT success and FMT failure. Notably, we did not find any explanation for efficacy based on the donor profiles. Using LEfSe (linear discriminant analysis effect size), no significant differences were observed in the composition of donors associated with success vs failure. We next compared recipient profiles and observed that failure to be colonized by the donor microbiota was significantly associated with higher baseline levels of several taxa belonging to the Gammaproteobacteria class of the Proteobacteria phylum (Fig. 6a).

Finally, we analyzed the overall population and looked for microbiota-based predictors of flare before the end of follow-up at 6 weeks post FMT or sham. We identified several taxa associated with flare, including many taxa belonging to the Gammaproteobacteria class and the Clostridiales order comprising *Ruminococcus gnavus*. In addition, we also observed taxa associated with maintenance of remission, such as *Ruminococcaceae*, *Coprococcus*, and *Desulfovibrio* genus (Fig. 6b).

## Discussion

In this pilot randomized controlled study, we evaluated the effect of a single FMT administered via colonoscopy in patients with colonic or ileo-colonic CD who achieved clinical remission with systemic corticosteroids. This is the first and only randomized controlled study evaluating FMT in CD to be published so far. Moreover, the original strategy we tested has never been evaluated in IBD.

Its primary endpoint, a > 60% similarity with the donor microbiota at week 6 according to the Sorensen similarity index, had been defined in 2012 without guidance from the recent literature. None of the subjects reached this endpoint, which however now appears inappropriate as some clinically relevant endpoints were achieved. Although not statistically significant, we observed a higher rate of steroid-free clinical remission in the FMT than in the sham group. We also observed significant benefit of FMT over sham with respect to CDEIS and CRP level. Notably, the absence of donor microbiota engraftment was associated with failure to maintain clinical remission.

Crohn’s disease, like UC, is thought to be related to an abnormal crosstalk between an over-activated intestinal immune system and an abnormal gut microbiota. Environmental and host genetic factors are also involved, and some of them may affect the disease through an effect on the gut microbiota. Indeed, several studies in mice have demonstrated that genetic defects in innate immunity genes [4, 27] or environmental factor [28] can induce alteration in gut microbiota composition and/or functions leading to deleterious effects on the host and favoring intestinal inflammation. Moreover, intestinal inflammation by itself induces perturbations in the composition of the gut microbiota [29]. These data suggest a circular causality between alterations to the gut microbiota and an over-activated immune system, fueled by environmental factors. The current treatments used in IBD do not take into account these new concepts, as they only target the immune actor in disease pathogenesis. FMT studies performed to date may encounter the same pitfall by targeting only the gut microbiota actor. In the current study, we tested for the first time a therapeutic approach targeting both the immune system and the microbiota by first shutting off inflammation with systemic corticosteroids, before reshaping the gut microbiota with FMT. We chose a relatively weak criterion (HBI < 5) to assess the corticosteroid-induced clinical remission as it represented only a step before initiating FMT or sham. This explains why we got a high response rate to corticosteroid. Although the number of subjects included was low, we think the results are of interest as they suggest that this strategy might be valid and may also give some insight into the dynamics of gut microbiota colonization following FMT.

Our data show a trend in favor of FMT regarding maintenance of remission. Although the delta between the FMT and sham groups regarding steroid-free clinical remission at week 10 was superior to 40%, the difference was not statistically significant, most likely because of a lack of power due to insufficient patient numbers. Nevertheless, we observed significant differences in terms of endoscopic activity and CRP level. Between day 0 and week 6, CDEIS decreased and CRP level remained stable in the FMT group, while CDEIS remained unchanged and CRP increased in the sham group, suggesting that inflammation was more successfully controlled in the FMT group. These results with regard to clinical efficacy are illuminated by the gut microbiota analysis. Firstly, we observed that in most of the patients, FMT is associated with an important and durable shift in the gut microbiota towards the donor’s microbiota. Surprisingly, we observed that the increase in alpha diversity was transient whereas the change in beta diversity was mostly maintained throughout the 6 months of follow-up. This suggests that some of the donor’s bacteria may colonize the patient in the long term. Secondly, we observed that two of the patients were not colonized by the donor’s microbiota, representing what we termed an “FMT failure.” These failures to colonize were not due to insufficient bowel cleansing in these patients, as it was judged perfect by the endoscopist on both occasions (Boston score 9/9). As some studies suggest a donor effect [16], we looked for differences between those associated with FMT failure vs FMT success but did not find any significant difference. Here again, these results should be taken cautiously as the number of patients was extremely low. We then looked for factors on the host side and observed that FMT failure was associated with enrichment in different members of the Gammaproteobacteria class (Proteobacteria phylum) such as *Klebsiella*, *Actinobacillus*, and *Haemophilus*. These results suggest that the baseline recipient microbiota might influence the success of donor microbiota colonization and consequently, the expected clinical outcome. This study has several limitations. The first one is its small size which requires to take its results cautiously. There were some non-significant differences at baseline in the two groups such as a lower CDEIS score in some patients of the sham group that might limit the possibility to observe a further decrease. Larger studies are needed to validate these results.

The relatively low similarity between donor and recipient microbiota at W6 suggest that a higher number of FMTs per patient and/or the use of multidonor infusions might be associated with better outcome, as suggested by other investigators in UC [17, 30].

## Conclusion

In this first randomized, sham-controlled pilot trial evaluating FMT in Crohn’s disease, the primary endpoint regarding donor microbiota colonization at week 6 was not achieved. The low similarity index between donor and recipient microbiota in some patients suggests that a single FMT might not be enough to induce significant changes in these patients. However, the benefit of FMT over sham transplantation was observed for several clinically relevant endpoints, including CDEIS and CRP level. These results need to be confirmed in larger studies. Several questions remain to be addressed. Can the current results be generalized to all CD patients and to UC? It is likely that if a “donor effect” exists, the study was underpowered to detect it. Many questions are also raised regarding the procedure, such as delivery mode, aerobic vs anaerobic stool preparation, the number of donors, and the need for single or repeated FMT? These questions will have to be addressed in larger studies in order to determine whether FMT is a viable therapeutic approach in common and long-term conditions such as IBD.

## Supplementary information

**Additional file 1:** Study design.

**Additional file 2.** Screening measures for donors.

**Additional file 3.** Raw sequence data.

**Additional file 4.** Characteristics of patients at selection (before starting corticosteroids.

**Additional file 5.** Change in clinical and biological parameters between day 0 and week 6 for FMT and sham treatment groups.

**Additional file 6.** (A) Sorensen similarity index between donor and recipient fecal microbiota 6 weeks after FMT or sham, separating “FMT” failure from “FMT success”.

**Additional file 7.** Principal coordinate analysis of Bray–Curtis distance in patients with FMT failure (A) or FMT sucess (B). Each PCoA plot represent the samples from a single patient across the different time points. PC1, PC2 and PC3 represent the top three principal coordinates that captured most of the diversity. The fraction of diversity captured by the coordinate is given as a percentage.

**Additional file 8.** Abundance of taxa at the genus/species level during the follow-up period in patients with FMT success (A) and FMT failure (B). Proportion of different OTU from donors in patients with FMT success and FMT failure (C). Only taxa representing > 0.1% of the microbiota were taken into account in the analysis.

**Additional file 9.** Evolution of similarity index (1-unweighted Unifrac) between donor and recipient fecal microbiota in patients with FMT success and FMT failure.

**Additional file 10.** Clinical efficacy of FMT is associated with colonization by the donor microbiota. Flare-free survival of patients in the FMT and the Sham groups.

## Data Availability

Raw sequence data are accessible in the European Nucleotide Archive (accession number PRJEB33031).
