# Supplementary figures and images for: Fecal microbiota transplantation to maintain remission in Crohn’s disease: a pilot randomized controlled study
Source: Microbiome. 2020 Feb 3;8:12. doi: 10.1186/s40168-020-0792-5 (PMC6998149; doi:10.1186/s40168-020-0792-5)

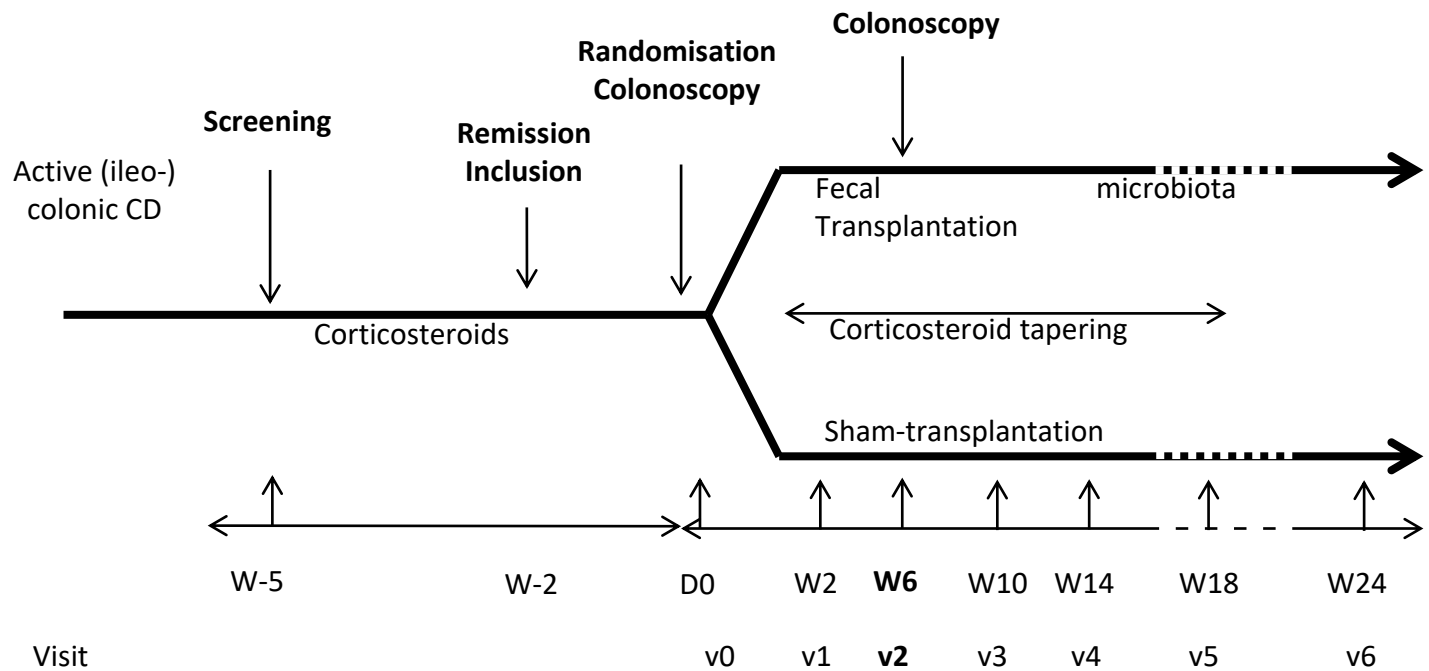

## Additional file 1: Study design.

Supplement: Supplementary file 2 — Additional file 1: Study design. [file 40168_2020_792_MOESM1_ESM.pdf]
