# Supplementary material for: Fecal microbiota transplantation to maintain remission in Crohn’s disease: a pilot randomized controlled study
Source: Microbiome. 2020 Feb 3;8:12. doi: 10.1186/s40168-020-0792-5 (PMC6998149; doi:10.1186/s40168-020-0792-5)
Supplement: Supplementary file 3 — Additional file 2. Screening measures for donors. [file 40168_2020_792_MOESM2_ESM.pdf]

## Additional file 2: Screening measures for donors

| Screening measure | Pathogen or condition investigated | | |
|---|---|---|---|
| **History collection via questionnaire** | **Medical history and clinical examination** | **Origin and Travel history** | **Behavior evaluation** |
| | 20 years < age < 50 years | Traveling to any area in the last 3 months* | Blood exposure accident |
| | 17 kg/m2 < Body Mass Index < 27 kg/m2 | Residence in intertropical area for several years | Tattoos or piercing in the last 6 months |
| | Blood transfusion in the last 6 months | Hospitalization outside France of the donor or a member of their family for > 24 hours in the last 12 months | Unsafe sexual behavior (intercourse without protection with a new partner in the preceding 6 months) |
| | Known infectious disease | | |
| | Viral infection in the 7 days before screening | | |
| | Acute gastroenteritis in the last 3 months | | |
| | Autoimmune or inflammatory disorders ** | | |
| | Gastrointestinal disease (IBD, irritable bowel syndrome, coeliac disease)** | | |
| | Gastrointestinal adenomas or neoplasm *** | | |
| | Hemorrhoid disease | | |
| | Presence of HSV and / or papilloma virus anal lesions | | |
| | History of typhoid fever infection | | |
| | Recent antibiotic/ antifungal treatment (last 3 months) | | |
| | Non-steroidal anti-inflammatory drugs (in the last 1 month) | | |
| | Immunosuppressant treatment, chemotherapy | | |
| | Chronic disease, long term treatment | | |
| | Pregnancy | | |
| | Vegetarian or specific diet | | |
| | Risk factors for Creutzfeldt-Jakob disease | | |
| | Recent intake of food allergens related to recipient's known allergy | | |
| Microbiological stool tests | **Bacteria** | **Parasites** | **Viruses** |
| | *Clostridium difficile* | Cyclospora / Isospora | Rotavirus |
| | *Campylobacter sp.* | Cryptosporidium / Microsporidium | Adenovirus |
| | *Shigella* | Entamoeba histolitica | Norvovirus |
| | *Yersinia* | Cryptospridium parvum | Enterovirus |
| | *Salmonella* | Anguillules | Poliovirus |
| | *Listeria monocytogenes* | Giardia intestinalis | Astrovirus |
| | *Vibrio cholerae/parahemolyticus* | Dientamoeba fragilis | Aichi virus, |
| | *Verotoxin-producing E. coli* | | Sapovirus |
| | Multi-drug resistant bacteria § | | Hepatitis A |
| Serological blood assays | **Bacteria** | **Parasites** | **Viruses** |
| | *Treponema Pallidum* | Strongyloides stercoralis | HIV |
| | | Trichinella spiralis | Hepatitis viruses (HAV, HBV, HCV, HEV) |
| | | Amoebiasis | HTLV1 and HTLV2 |
| | | Toxoplasma gondii §§ | EBV, CMV §§ |

* Except Euro area, United Kingdom, Bulgaria, Poland, Romania, Croatia, Hungary, Czech Republic, Denmark, Norway, Sweden, Swiss, USA or Canada

** Personal history or first degree relative

*** Personal history or first degree relative before 60 years of age

§ Enterobacteria producing extended spectrum betalactamase, Actinobacter baumanii, Vancomycin resistant enterococci and carbapenemase producing bacteria

§§ Sero-compatibility between patients and donor required
