# Supplementary material for: Fecal microbiota transplantation to maintain remission in Crohn’s disease: a pilot randomized controlled study
Source: Microbiome. 2020 Feb 3;8:12. doi: 10.1186/s40168-020-0792-5 (PMC6998149; doi:10.1186/s40168-020-0792-5)
Supplement: Supplementary file 5 — Additional file 4. Characteristics of patients at selection (before starting corticosteroids. [file 40168_2020_792_MOESM4_ESM.pdf]

Additional file 4 : Characteristics of patients at selection (before starting corticosteroids)

	Whole population (n=17)	Sham transplantation (n=9)	Fecal transplantation (n=8)	Pvalue (Sham vs FMT)
**Hemoglobin (g/L)**	130 [120 ; 140]	131 [120 ; 140]	129 [119 ; 141]	0.35
**White cell count (10^9/L)**	9.5 [8.4 ; 11.7]	9.4 [7.9 ; 10.8]	10.9 [8.8 ; 12.4]	0.22
**Platelet** (10^9/L)	337 [295 ; 407]	354 [316 ; 383]	312 [287 ; 482]	0.44
**CRP (mg/L)**	10.8 [5.0 ; 35.4]	9.4 [6.2 ; 39.3]	13.3 [2.5 ; 34.7]	0.33
**HBI Score**	7.0 [6.0 ; 9.0]	8.0 [7.0 ; 9.0]	6.0 [5.75 ; 6.25]	0.02
**Current azathioprine treatment**	4 (23.5)	2 (22.2)	2 (25.0)	0.89

CRP: C reactive protein; HBI : Harvey Bradshaw Index. Categorical parameters indicated as n (%) and continuous values indicated as P50 [P25 ; P75]. Quantitative variables were compared using a non–parametric Wilcoxon rank sum tests. Qualitative variables were compared using Fisher exact test.
