# Supplementary material for: Fecal microbiota transplantation to maintain remission in Crohn’s disease: a pilot randomized controlled study
Source: Microbiome. 2020 Feb 3;8:12. doi: 10.1186/s40168-020-0792-5 (PMC6998149; doi:10.1186/s40168-020-0792-5)
Supplement: Supplementary file 6 — Additional file 5. Change in clinical and biological parameters between day 0 and week 6 for FMT and sham treatment groups. [file 40168_2020_792_MOESM5_ESM.pdf]

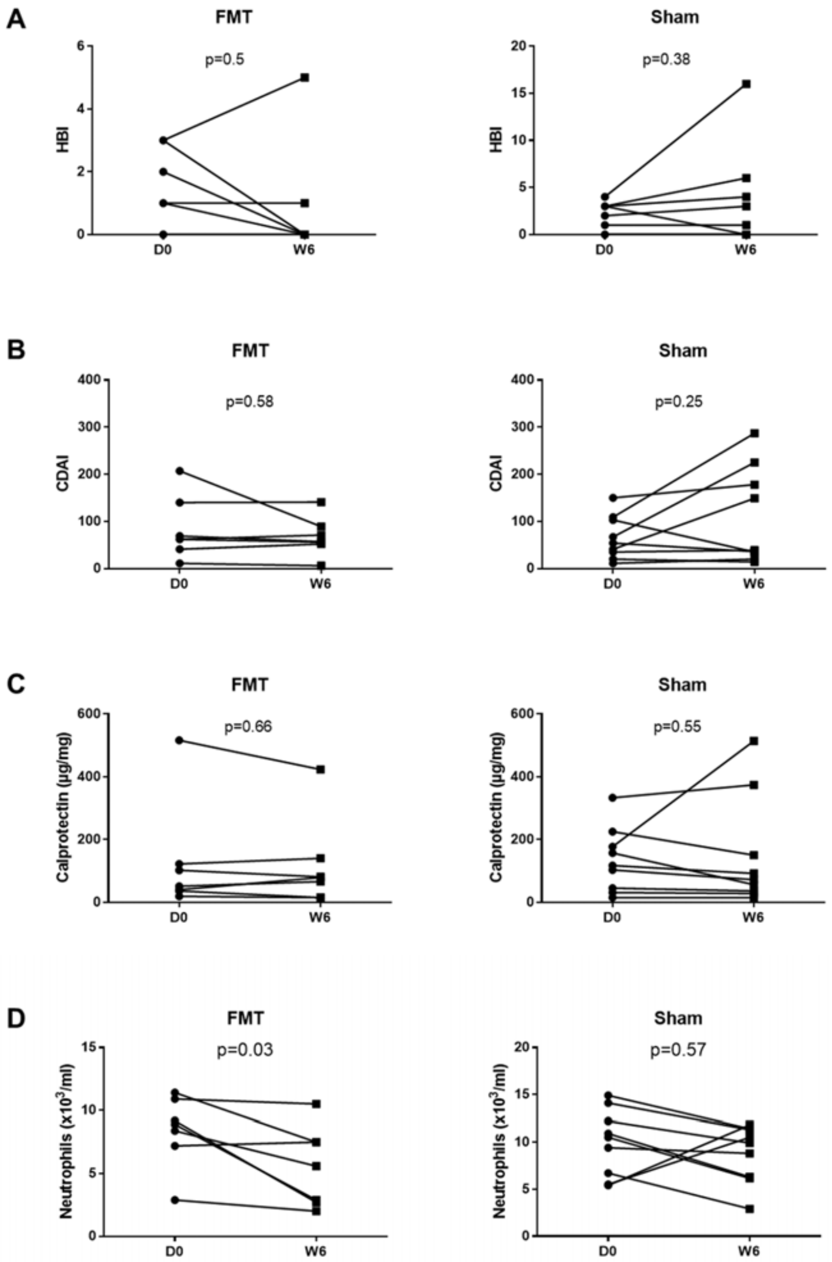


**Additional File 5: Change in clinical and biological parameters between day 0 and week 6 for FMT and sham treatment groups**. **(A)** HBI, **(B)** CDAI, **(C)** fecal calprotectin, **(D)** neutrophil counts. Comparisons evaluated with paired Wilcoxon test. One patient in each group was not evaluable for CDEIS because of a bowel-cleansing problem at week 6. One sample was not available in the FMT group at week 6.
