# Supplementary material for: Fecal microbiota transplantation to maintain remission in Crohn’s disease: a pilot randomized controlled study
Source: Microbiome. 2020 Feb 3;8:12. doi: 10.1186/s40168-020-0792-5 (PMC6998149; doi:10.1186/s40168-020-0792-5)
Supplement: Supplementary file 7 — Additional file 6. (A) Sorensen similarity index between donor and recipient fecal microbiota 6 weeks after FMT or sham, separating “FMT” failure from “FMT success”. [file 40168_2020_792_MOESM6_ESM.pdf]

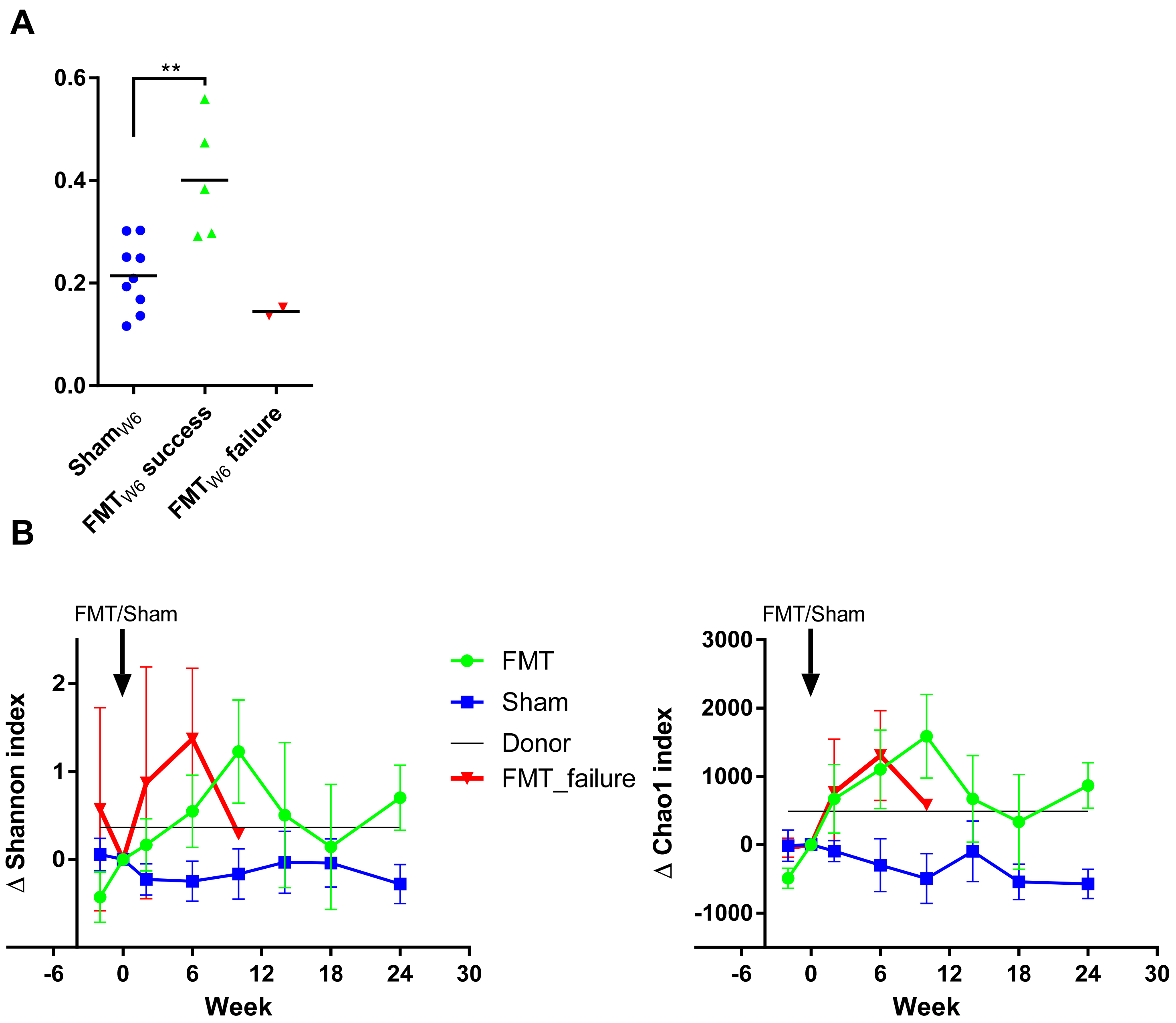

Additional File 6: (A) Sorensen similarity index between donor and recipient fecal microbiota 6 weeks after FMT or sham, separating "FMT" failure from "FMT success". For the Sham group, the mean of the Sorensen with each donor was indicated. **(B)** Change in Shannon and Chao1 alpha diversity indices compared to day 0 and according FMT failure or success. For donor, change was calculated with mean of FMT group. For A, Wilcoxon rank sum test was used. *: $p<0.05$; **: $p<0.01$.
