# Supplementary material for: Fecal microbiota transplantation to maintain remission in Crohn’s disease: a pilot randomized controlled study
Source: Microbiome. 2020 Feb 3;8:12. doi: 10.1186/s40168-020-0792-5 (PMC6998149; doi:10.1186/s40168-020-0792-5)
Supplement: Supplementary file 8 — Additional file 7. Principal coordinate analysis of Bray–Curtis distance in patients with FMT failure (A) or FMT sucess (B). Each PCoA plot represent the samples from a single patient across the different time points. PC1, PC2 and PC3 represent the top three principal coordinates that captured most of the diversity. The fraction of diversity captured by the coordinate is given as a percentage. [file 40168_2020_792_MOESM7_ESM.pdf]

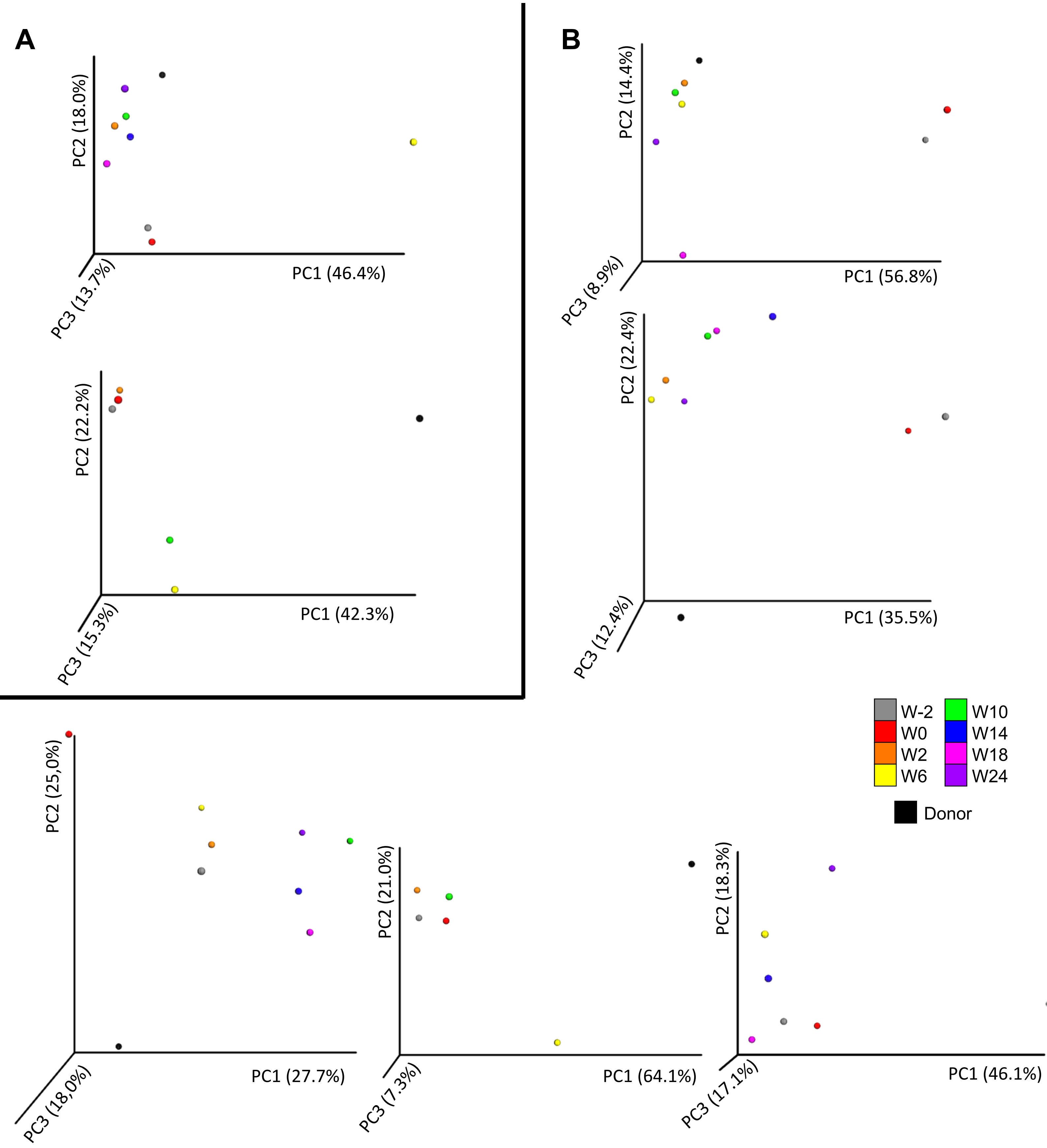

Additional File 7: Principal coordinate analysis of Bray–Curtis distance in patients with FMT failure (**A**) or FMT sucess (**B**). Each PCoA plot represent the samples from a single patient across the different time points. PC1, PC2 and PC3 represent the top three principal coordinates that captured most of the diversity. The fraction of diversity captured by the coordinate is given as a percentage.
