# Supplementary material for: Fecal microbiota transplantation to maintain remission in Crohn’s disease: a pilot randomized controlled study
Source: Microbiome. 2020 Feb 3;8:12. doi: 10.1186/s40168-020-0792-5 (PMC6998149; doi:10.1186/s40168-020-0792-5)
Supplement: Supplementary file 9 — Additional file 8. Abundance of taxa at the genus/species level during the follow-up period in patients with FMT success (A) and FMT failure (B). Proportion of different OTU from donors in patients with FMT success and FMT failure (C). Only taxa representing > 0.1% of the microbiota were taken into account in the analysis. [file 40168_2020_792_MOESM8_ESM.pdf]

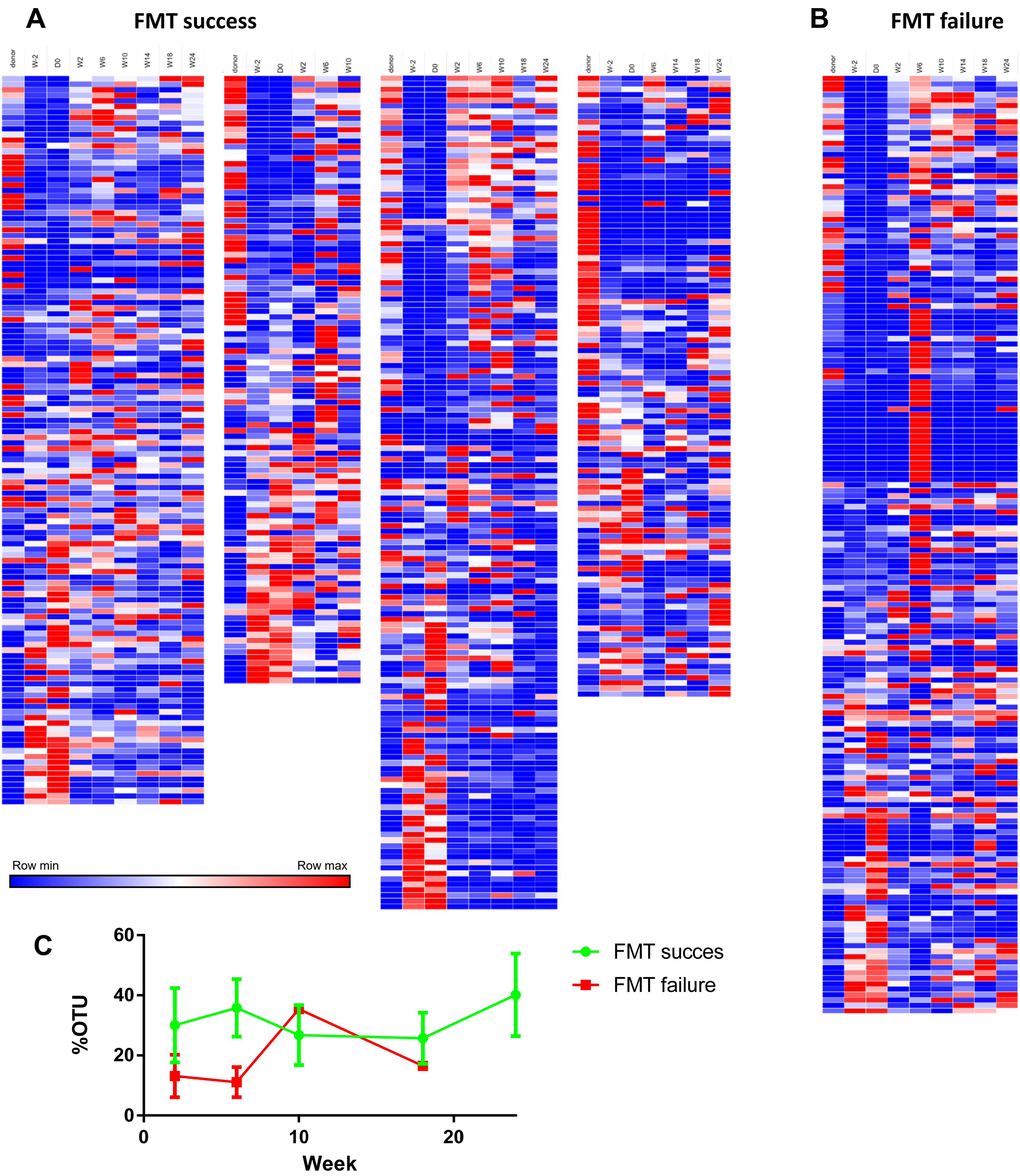

Additional File 8: Abundance of taxa at the genus/species level during the follow-up period in patients with FMT success (**A**) and FMT failure (**B**). Proportion of different OTU from donors in patients with FMT success and FMT failure (**C**). Only taxa representing > 0.1% of the microbiota were taken into account in the analysis.
