# Supplementary material for: Fecal microbiota transplantation to maintain remission in Crohn’s disease: a pilot randomized controlled study
Source: Microbiome. 2020 Feb 3;8:12. doi: 10.1186/s40168-020-0792-5 (PMC6998149; doi:10.1186/s40168-020-0792-5)
Supplement: Supplementary file 10 — Additional file 9. Evolution of similarity index (1-unweighted Unifrac) between donor and recipient fecal microbiota in patients with FMT success and FMT failure. [file 40168_2020_792_MOESM9_ESM.pdf]

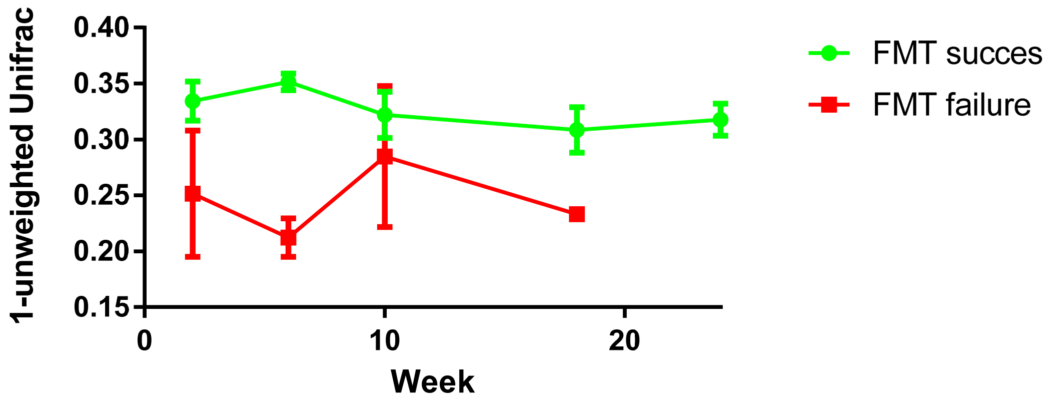

Additional File 9: Evolution of similarity index (1-unweighted Unifrac) between donor and recipient fecal microbiota in patients with FMT success and FMT failure.
