# Supplementary material for: Fecal microbiota transplantation to maintain remission in Crohn’s disease: a pilot randomized controlled study
Source: Microbiome. 2020 Feb 3;8:12. doi: 10.1186/s40168-020-0792-5 (PMC6998149; doi:10.1186/s40168-020-0792-5)
Supplement: Supplementary file 11 — Additional file 10. Clinical efficacy of FMT is associated with colonization by the donor microbiota. Flare-free survival of patients in the FMT and the Sham groups. [file 40168_2020_792_MOESM10_ESM.pdf]

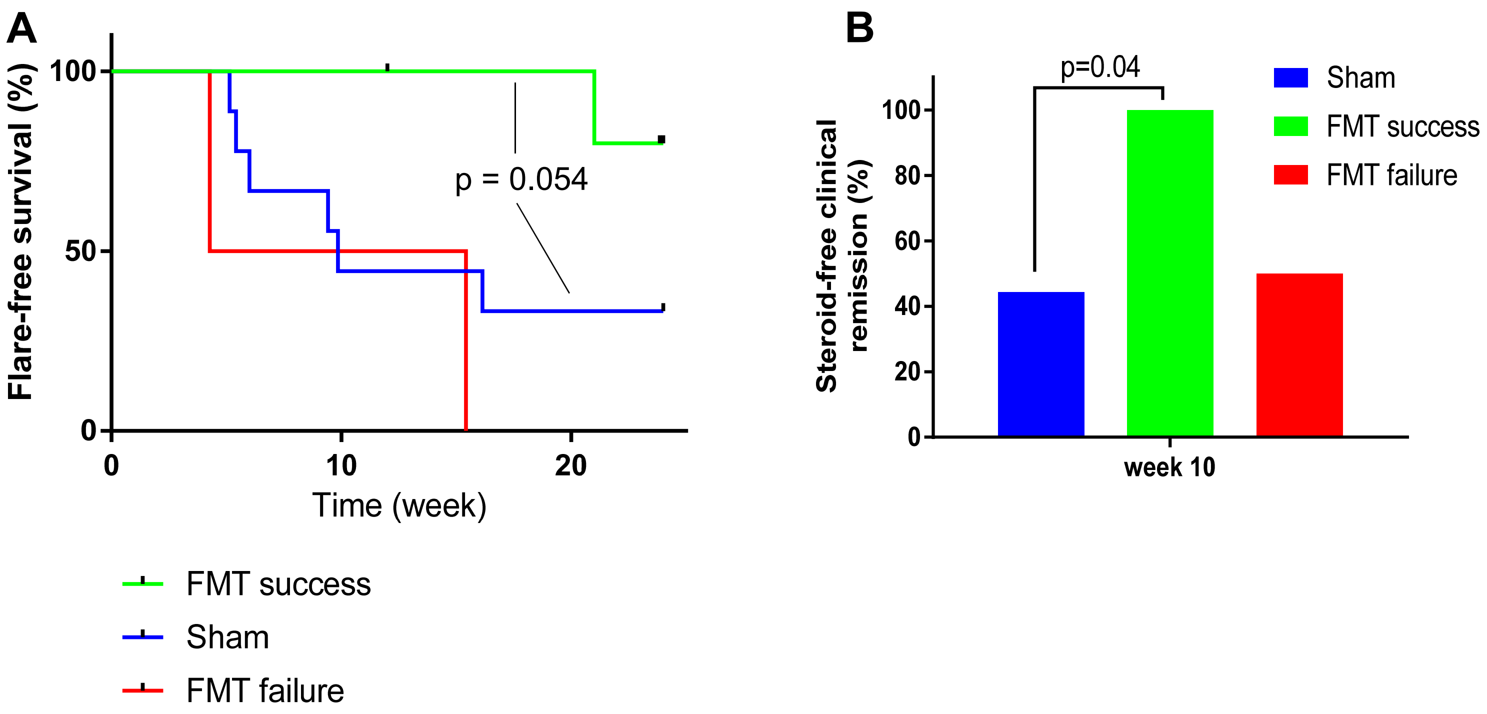

Additional File 10: Clinical efficacy of FMT is associated with colonization by the donor microbiota. Flare-free survival of patients in the FMT and the Sham groups. FMT success and Sham groups were compared using the logrank test. **(B)** Steroid-free remission at week 10 after FMT or Sham transplantation. FMT success and Sham groups were compared using Fisher's exact test.
